# Five-fraction SBRT for ultra-central NSCLC in-field recurrences following high-dose conventional radiation

**DOI:** 10.1186/s13014-017-0897-6

**Published:** 2017-10-19

**Authors:** Michael C. Repka, Nima Aghdam, Shaan K. Kataria, Lloyd Campbell, Simeng Suy, Sean P. Collins, Eric Anderson, Jonathan W. Lischalk, Brian T. Collins

**Affiliations:** 1 Department of Radiation Medicine, Georgetown University Hospital, 3800 Reservoir Road NW, Washington, DC 2007 USA; 2 Division of Pulmonary, Critical Care, and Sleep Medicine, Georgetown University Hospital, 3800 Reservoir Road NW, Washington, DC 2007 USA

**Keywords:** Sbrt, Reirradiation, Nsclc, Lung cancer, Ultra-central

## Abstract

**Purpose/objective:**

Local treatment options for patients with in-field non-small cell lung cancer (NSCLC) recurrence following conventionally fractionated external beam radiation therapy (CF-EBRT) are limited. Stereotactic body radiation therapy (SBRT) is a promising modality to achieve reasonable local control, although toxicity remains a concern.

**Materials/methods:**

Patients previously treated with high-dose CF-EBRT (≥59.4 Gy, ≤3 Gy/fraction) for non-metastatic NSCLC who underwent salvage SBRT for localized ultra-central in-field recurrence were included in this analysis. Ultra-central recurrences were defined as those abutting the trachea, mainstem bronchus, or esophagus and included both parenchymal and nodal recurrences. The Kaplan-Meier method was used to estimate local control and overall survival. Durable local control was defined as ≥12 months. Toxicity was scored per the CTC-AE v4.0.

**Results:**

Twenty patients were treated with five-fraction robotic SBRT for ultra-central in-field recurrence following CF-EBRT. Fifty percent of recurrences were adenocarcinoma, while 35% of tumors were classified as squamous cell carcinoma. The median interval between the end of CF-EBRT and SBRT was 23.3 months (range: 2.6 – 93.6 months). The median CF-EBRT dose was 63 Gy (range: 59.4 – 75 Gy), the median SBRT dose was 35 Gy (range: 25 – 45 Gy), and the median total equivalent dose in 2 Gy fractions (EQD2) was 116 Gy (range: 91.3 – 136.7 Gy). At a median follow-up of 12 months for all patients and 37.5 months in surviving patients, the majority of patients (90%) have died. High-dose SBRT was associated with improved local control (*p* < .01), and the one-year overall survival and local control were 77.8% and 66.7% respectively in this sub-group. No late esophageal toxicity was noted, although a patient who received an SBRT dose of 45 Gy (total EQD2: 129.7 Gy) experienced grade 5 hemoptysis 35 months following treatment.

**Conclusions:**

Although the overall prognosis for patients with in-field ultra-central NSCLC recurrences following CF-EBRT remains grim, five-fraction SBRT was well tolerated with an acceptable toxicity profile. Dose escalation above 35 Gy may offer improved local control, however caution is warranted when treating high-risk recurrences with aggressive regimens.

## Introduction

Although the incidence of lung and bronchus cancer has been steadily decreasing in the United States, the disease is still responsible for more deaths per year than any other malignancy [1]. While patients with early stage non-small cell lung cancer (NSCLC) have seen continuous expansion and improvement in available treatment options, the prognosis for patients with locally advanced disease is poor and often presents an oncologic dilemma to the treating physician. Frequently patients are not good candidates for surgical resection due to disease extent or medical comorbidity [2], and radiation therapy plays a key role in patient management, frequently employing doses of 60 Gy or more [3, 4]. Survival rates for patients with locally advanced NSCLC reported in the literature are grim, with an estimated 5-year overall survival for stage IIIA and IIIB patients of 19% and 7% respectively according to the 2007 International Association for the Study of Lung Cancer (IASLC) database analysis [5].

As innovations in systemic therapy, surgery, and radiation techniques are implemented, the prognosis for those patients with advanced disease should improve. According to the 2016 update of the IASLC database analysis, 5-year overall survival rates have soared over the past decade to 36% and 19% in stage IIIA and IIIB patients respectively [6]. While such a drastic improvement in 5-year overall survival for locally advanced patients must be taken with a degree of caution, these data are highly encouraging. However, local recurrence is a common problem in this patient population [4], and treatment options for patients with recurrent NSCLC who have previously undergone high-dose thoracic radiation are extremely limited [7–10], particularly when disease is situated within the previously treated portal. Given increasing patient longevity, the gravity of preventing morbid local failure may grow ever more paramount.

Newer modalities of radiation therapy, such as stereotactic body radiation therapy (SBRT) and proton beam therapy (PBT), may allow for safer retreatment of previously irradiated tissue by limiting radiation dose to normal tissue and organs-at-risk (OARs). Furthermore, the high dose-per-fraction typically employed with SBRT may provide a higher degree of therapeutic efficacy given the radioresistance of many lung cancers, particularly those that have already received high doses of conventionally fractionated radiation [11]. While there is considerable accumulating evidence regarding the role of SBRT for early-stage lung cancers [12, 13], there are few studies which have examined its role in the management of previously irradiated recurrent NSCLC [14–21]. However, these retrospective, single-institution studies do suggest the relative safety of SBRT reirradiation for peripheral lesions.

Definitive SBRT for lesions within 2 cm of the central airway in the unirradiated chest has been associated with higher toxicity than for peripheral treatments [22]. Furthermore, a 15% rate of fatal pulmonary hemorrhage has been reported following hypofractionated treatment of ultra-central tumors, in which the target volume overlapped with central airways [23]. One hypothesis for the enhanced morbidity of central treatment is the radiosensitivity of bronchial cartilage, which is thought to be sensitive to the high dose-per-fraction schema typically employed with SBRT, and indeed fatal central airway necrosis has been observed in these patients [24]. Additionally, late esophageal toxicity, such as stricture or fistula, has been observed with thoracic SBRT [25, 26].Consequently, recurrent central lesions must be considered exquisitely high-risk when they are situated within the prior radiation field, as is common following either adjuvant or definitive treatment for locally advanced NSCLC. While SBRT may be feasible for retreatment of such lesions, the optimal dose to achieve durable local control without excessive toxicity is unclear. In this study, we retrospectively review our institutional experience using SBRT to reirradiate ultra-central NSCLC recurrences previously treated with high dose conventionally fractionated external beam radiation therapy (CF-EBRT).

## Materials & methods

### Patient selection

The Georgetown University Institutional Review Board (IRB) approved this single institution retrospective study (IRB #2013-0801). Patients eligible for study inclusion had a history of pathologically confirmed locally advanced NSCLC which was treated with radical CF-EBRT and subsequently suffered an ultra-central local recurrence within the previously irradiated field. Ultra-central local recurrence was defined as a lesion located within the prior planning treatment volume (PTV) or radiation portals that was centered within either the lung parenchyma, hilum, or mediastinum with direct abutment of the trachea, mainstem bronchus, or esophagus as visualized on computed-tomography (CT) scan. Pathologic confirmation of recurrence was not required for study inclusion. Criteria for high-dose CF-EBRT consisted of a minimum total prescription dose of 59.4 Gy with a maximum daily dose of 3 Gy utilizing conventional radiotherapy, 3D conformal radiotherapy (3D-CRT), or intensity modulated radiation therapy (IMRT). Only patients with ‘local-only’ failure were included in the analysis; those patients with concurrent distant metastatic disease or out-of-field failures were excluded. Patients whose local recurrence was treated with robotic SBRT in five-fractions were included in the analysis.

#### Radiation treatment planning and delivery

Patients were simulated in the supine position with their arms at their side. All patients underwent high-resolution deep-inspiration CT scan with 1.25 mm slice thickness. Four-dimensional CT (4D-CT) scan was not obtained. The recurrent gross tumor volume (rGTV) and OARs were contoured in concert by the same treating radiation oncologist (BTC) and interventional pulmonologist (EA). Radiation dose was prescribed directly to the rGTV; no clinical tumor volume (CTV) or PTV expansions were applied. Treatment plans were generated using inverse planning in MultiPlan (Accuray Inc., Sunnyvale, CA, USA) and typically consisted of more than 100 of non-isocentric pencil beams.

**Fig. 1:**
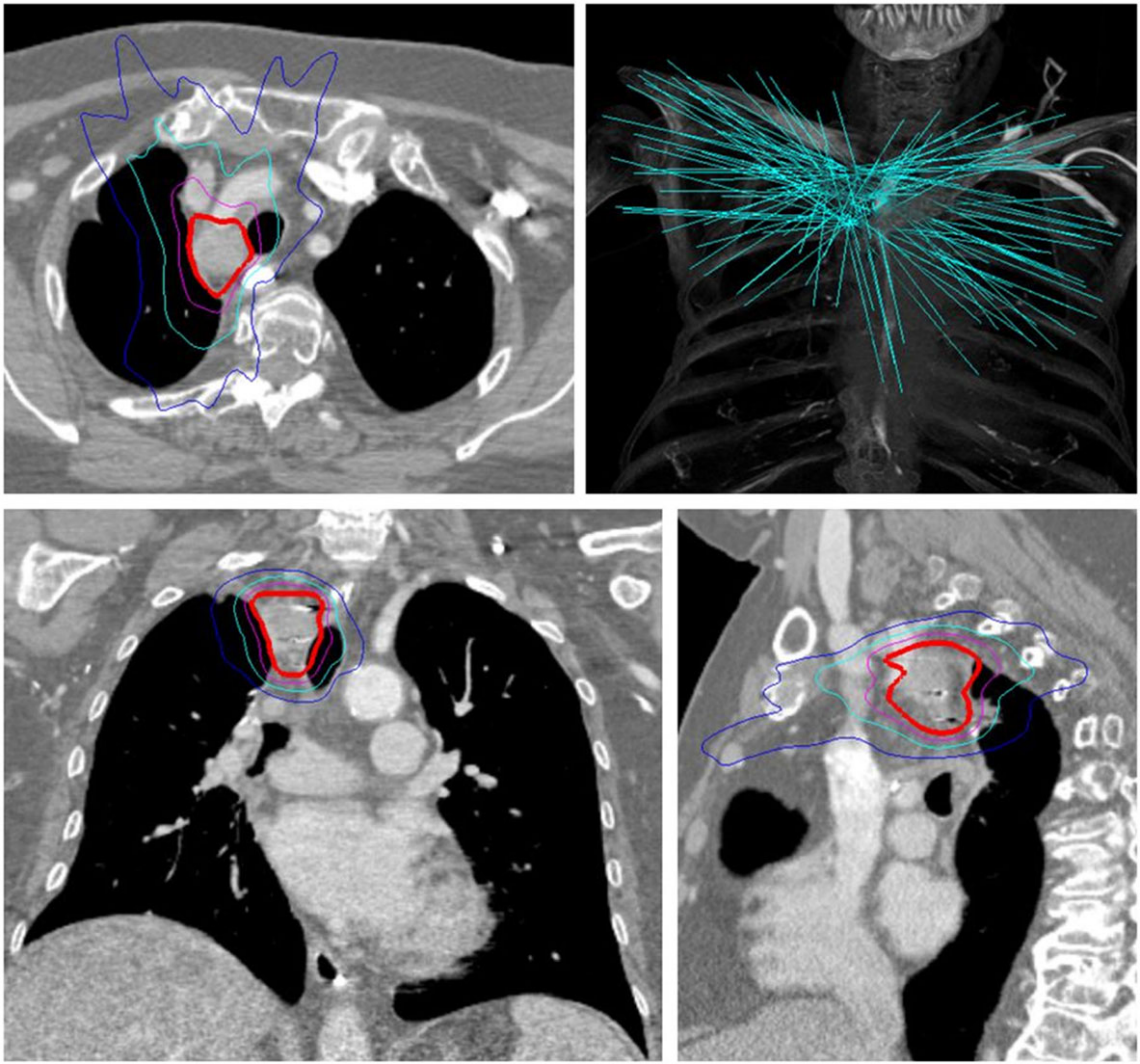
Sample Treatment Plan: axial, sagittal, and coronal views. Red line indicates the prescription isodose line (40 Gy). Purple line indicates the 30 Gy isodose line, cyan line indicates the 20 Gy isodose line, and blue line indicates the 10 Gy isodose line. Robotic SBRT non-isocentric treatment beams are illustrated in the upper right-hand corner

Radiation treatment was delivered in five fractions using the CyberKnife Robotic Radiosurgery System (Accuray Inc., Sunnyvale, CA, USA). Motion of the rGTV was tracked via paired orthogonal X-ray acquisition and matching during treatment, utilizing Xsight Spinal Tracking (Accuray Inc., Sunnyvale, CA, USA) when fiducial tracking was not available. The Synchrony Respiratory Motion Tracking system (Accuray Inc., Sunnyvale, CA, USA) was employed to account for rGTV movement throughout the respiratory cycle in patients treated with fiducial markers. No formal size limitations for the rGTV were utilized, and normal structure constraints were considered on a case-by-case basis by the treating radiation oncologist (BTC). Total SBRT dose was stratified as low (<40 Gy) or high (≥40 Gy). A representative treatment plan is illustrated in Fig. 1.

#### Follow-up and statistical analyses

No formal follow-up guidelines were utilized to assess treatment response, but in general patients underwent follow-up CT scan at 3 month intervals or as clinically warranted per institutional practice. Local failure was scored when local progression of disease was evident on CT or positron-emission tomography (PET) scan as determined by the treating radiation oncologist and pulmonologist. If local recurrence was felt to be equivocal at a given follow-up point, but later confirmed to be recurrence, the time of local failure was back-dated to the initial equivocal finding. For patients who experienced progression of local disease at the first follow-up imaging, they were considered to have never achieved local control. Durable local control was defined as lack of disease progression for a minimum of 12 months following completion of treatment.

The Kaplan-Meier method was used to estimate local control and overall survival from the time of SBRT conclusion. The equivalent dose in 2 Gy fractions (EQD2) was calculated from the nominal conventional and SBRT prescription doses using an α/β ratio of 10. Total EQD2 was determined by combining the conventional and SBRT doses. Statistically significant predictors of overall survival and freedom from local progression were identified using the log-rank test. Differences in mean interval between CF-EBRT and SBRT were analyzed using the Student’s *t-*test. Late toxicity was scored according to the National Cancer Institute Common Terminology Criteria for Adverse Events, Version 4.0 (NCI-CTCAE v4.0). After stratification by SBRT dose, differences in mean values of continuous variables were analyzed using the Student’s *t-*test, and proportional differences in categorical variables were assessed using Fisher’s exact test. Statistical analysis was performed using both Excel for Mac 2011 (Microsoft Corporation, Redmond, WA, USA) and SPSS Statistics version 23.0 (IBM Corporation, Armonk, NY, USA).

## Results

### Patient and radiation therapy details

Between November 2004 and August 2014, a total of 20 patients were identified meeting study entry criteria. The median patient age was 70.5 years, with a range of 47 to 90 years. Twelve patients were male (60%), and eight patients were female (40%). The median Eastern Cooperative Oncology Group (ECOG) performance status score was 0 (range: 0 – 3). The most common histology was adenocarcinoma (*n* = 10, 50%), followed by squamous cell carcinoma (*n* = 7, 35%). Other histologies identified included large cell carcinoma, undifferentiated carcinoma, and adenosquamous carcinoma (one each). Twelve lesions were centered in the mediastinum, with the most common location in an upper or lower paratracheal lymph node station (*n* = 9). Five lesions were located in the hilum, two lesions were centered within the upper lobe of the lung, one lesion was located within multiple mediastinal nodal stations, and a single lesion was centered in the subcarinal area (station 7). The median interval between completion of CF-EBRT and initiation of SBRT reirradiation was 30.8 months (range: 2.6 – 93.6). In patients for whom PET data was available (*n* = 15), the mean maximum standardized uptake value (SUV) was 11.8 (range: 7.0 – 30.4). Twelve patients (60%) received sequential chemotherapy with SBRT re-irradiation, while the remainder were treated with SBRT alone. Full baseline patient characteristics are presented in Table 1.

**Table 1:** Baseline patient characteristics

Characteristic	Median (Range) / Number (%)
Age	70.5 (47 – 90)
Treatment Interval (months)	30.8 (2.6 – 93.6)
Gender	
Male	12 (60%)
Female	8 (40%)
ECOG PS	
0	12 (60%)
1	5 (25%)
2	1 (5%)
3	2 (10%)
Histology	
Adenocarcinoma	10 (50%)
Squamous Cell Carcinoma	7 (35%)
Other	3 (15%)
Lesion Location	
Mediastinum	10 (50%)
Hilum	8 (40%)
Upper Lobe	2 (10%)
Sequential Chemotherapy^a^	
Yes	12 (60%)
No	8 (40%)
Recurrence SUV Max	11.8 (7.0 – 34.0)

^a^Given either before or after SBRT at the time or recurrence

The median prior CF-EBRT prescription dose was 63.0 Gy (range: 59.4 – 75), with a median daily dose of 1.8 Gy (range: 1.8 – 2.5). Using an α/β ratio of 10 Gy, the corresponding median CF-EBRT EQD2 was 62.0 Gy (range: 58.4 – 78.1). The median rGTV was 79.6 cm^3^ (range: 6.0 – 318.0), and the median SBRT re-irradiation prescription dose was 35 Gy (range: 25 Gy – 45 Gy), with all patients treated in five fractions. The median prescription isodose line was 77% (range: 65% – 83%), with a median maximum plan point dose of 48.6 Gy (range: 32.1 – 57.0) and a median maximum esophageal dose of 25.4 Gy (range: 5.9 – 36.6). The median cumulative prescription EQD2 was 116.0 Gy (range: 91.3 – 136.7). Full details of SBRT re-irradiation are presented in Table 2.

**Table 2:** Radiation treatment details

Characteristic	Median (Range)
CF-EBRT Treatment	
Prescription Dose (Gy)	63.0 (59.4 – 75.0)
Daily Dose (Gy)	1.8 (1.8 – 2.5)
EQD2 (Gy)	62.0 (58.4 – 78.1)
SBRT Reirradiation	
Recurrent GTV (cc)	79.6 (6.0 – 318.0)
Prescription IDL (%)	77.0 (65.0 – 83.0)
Prescription Dose (Gy)	35.0 (25.0 – 45.0)
Daily Dose (Gy)	7.0 (5.0 – 9.0)
Maximum Point Dose (Gy)	48.6 (32.1 – 57.0)
Maximum Esophageal Dose (Gy)	25.4 (5.9 – 36.6)
Cumulative EQD2 (Gy)	116.0 (91.3 – 136.7)

When stratified by SBRT dose, there were no significant differences in patient or recurrent tumor characteristics (Table 3). Although there was a trend toward larger recurrent tumors in the low-dose SBRT arm (147.06 cm^3^ vs. 71.36 cm^3^), this did not reach the level of statistical significance (*p* = 0.068). In the low dose arm, three patients received 25 Gy, four patients received 30 Gy, and four patients received 35 Gy. In the high dose arm, seven patients received 40 Gy, while two patients received 45 Gy. No significant differences were noted with regard to available re-irradiation plan data, with the exception of maximum SBRT plan point dose, SBRT EQD2, and total tumor EQD2.

**Table 3:** Patient and tumor characteristics stratified by radiation dose

Variable	Mean / Number		*p-*value
	<40 Gy	≥40 Gy	
Age (Years)	66.36	72.44	0.230
GTV (cc)	147.06	71.36	0.068
Esophagus Max Point Dose (Gy)	25.74	21.61	0.337
Spinal Cord Max Point Dose (Gy)	9.61	8.26	0.473
SBRT Plan Max Point Dose (Gy)	41.71	51.86	<0.01
Prior Dose (Gy)	63.91	62.67	0.481
Prior EQD2 (Gy)	63.27	61.97	0.526
Tumor SBRT EQD2 (Gy)	41.10	62.50	<0.01
Total Combined EQD2 (Gy)	104.36	124.47	<0.01
Gender			
Male	7	5	0.535
Female	4	4	
ECOG PS			
0	8	4	0.289
1	3	2	
2	0	1	
3	0	2	
Chemotherapy			
Yes	3	5	0.362
No	8	4	

### Local control and overall survival

**Fig. 2:**
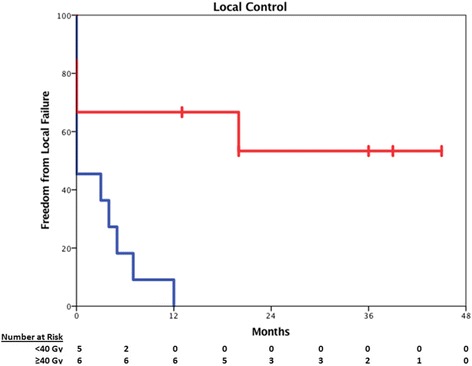
Local Control. Red line indicates LC in patients who received at least 40 Gy, while the blue line indicates those patients receiving less than 40 Gy (Log-rank *p* < 0.01). Censored patients are indicated by hash marks

At a median follow-up of 12 months for all patients and 37.5 months in surviving patients, the majority of patients (90%) have died. The one-year local control was 30% in all patients, although a significant proportion of patients never achieved local control (45%). However, the 1 year-local control estimate for patients who received high-dose SBRT (≥40 Gy, *n* = 9) was significantly improved (66.7% vs. 0.0%, *p* < 0.01, Fig. 2). Of these patients in the high-dose SBRT strata who achieved durable local control, only a single patient suffered local recurrence prior to death. Furthermore, an increased mean interval between initial treatment and SBRT was observed in patients who achieved durable local control (41.9 vs. 13.4 months, p < 0.01).

**Fig. 3:**
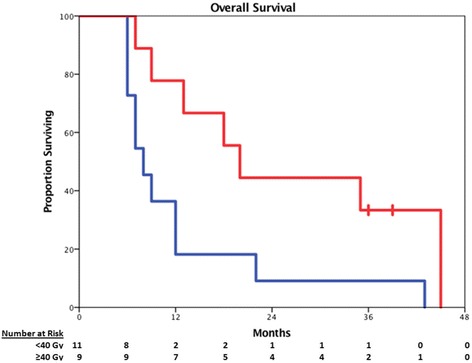
Overall Survival. Red line indicates OS in patients who received at least 40 Gy, while the blue line indicates those patients receiving less than 40 Gy (Log-rank *p* = 0.026). Censored patients are indicated by hash marks

The one-year overall survival estimate for the entire cohort was 45%, with a median survival of 12 months. However, high-dose SBRT was associated with improved median overall survival compared to patients who received less than 40 Gy (20.0 months vs. 8.0 months, *p* = .026, Fig. 3), and the one-year overall survival was 77.8% in this sub-group. There was no difference in mean interval between initial treatment and SBRT in patients who survived at least 12 months compared to those who did not (23.3 vs. 16.0 months, *p* = 0.40).

### Toxicity

While treatment was generally well tolerated, there were two cases of radiation pneumonitis (grade 2) and two cases of recurrent laryngeal nerve paralysis (grades 2 and 3), all of which resolved prior to last follow-up or death. Comprehensive dosimetry data was not available from initial CF-EBRT plans for several patients, and no specific cumulative EQD2 predictors of pulmonary or neural toxicity could be identified. Considering dosimetric data from SBRT plans alone, no significant predictors of pulmonary toxicity could be identified, though no dosimetry data was available for the recurrent laryngeal nerve. No patient exhibited late esophageal toxicity. A single patient who received an SBRT dose of 45 Gy (cumulative EQD2: 129.7 Gy) experienced sudden death 35 months following treatment which was attributed to radiation toxicity (hemoptysis), although the cause of death was not formally proven. Individual patient toxicities are summarized in Table 4.

**Table 4:** Individual Patient Late Grade 2+ Toxicity

Toxicity	SBRT Dose (Gy)	Cumulative EQD2 (Gy)	Grade	Treatment	Resolution
Pneumonitis	35.0	109.8	2	Oral Prednisone	Yes
Pneumonitis	40.0	120.0	2	Oral Prednisone	Yes
Recurrent Laryngeal Nerve	40.0	122.0	2	None	Yes
Recurrent Laryngeal Nerve	40.0	125.5	3	Vocal Cord Injection	Yes
Hemoptysis	45.0	129.7	5	None	Death

## Discussion

For patients with in-field recurrence of NSCLC following high-dose CF-EBRT, the prognosis is generally poor. Treatment options are limited and frequently ineffective [7–10], particularly in a patient population with a high incidence of medical comorbidity. In our single-institution study, we report a median survival of 1 year, which is consistent with the limited available literature. However, when patients were stratified by dose, we observed a wide divergence in meaningful clinical outcomes. Patients who received less than 40 Gy experienced significantly worse local control, and no patient in this sub-group achieved durable local control. This finding is consistent with existing evidence for SBRT dose response in the definitive setting, where improved local control is seen with biologically effective doses (BEDs) greater than 100 Gy [27]. More surprisingly, a similar phenomenon was observed when considering patient survival, as dose escalation was associated with improved patient survival. These findings can potentially be explained by several factors. First, given the central nature of these lesions, local failure would likely be associated with rapid patient mortality given the proximity of critical organs such as the heart, lungs, and airways. Second, we excluded patients with metastatic disease, and local control may be critical in the cohort of patients for whom systemic death is not imminent.

Although emerging data suggest the safety and tolerability of SBRT reirradiation following high-dose thoracic CF-EBRT (Table 5), many of these studies comprise patients with peripheral or out-of-field recurrences. In 2014, Trovo et al. reported their experience with SBRT reirradiation of in-field central recurrences, which were defined in as those within 2 cm of the proximal tracheobronchial tree. In our study, criteria for study inclusion were more strict – ultra-central tumors were required to have evidence of major airway or esophageal abutment on CT scan. Several other important differences exist between these studies, which include target volume delineation, prescription dose, and radiation technique. Notably, Trovo et al. employed a 5 mm isotropic expansion to the GTV in order to create a PTV, whereas in our report the dose was prescribed directly to the GTV. Given the similar rates of local control in their patients, who received 30 Gy in 5 to 6 fractions prescribed to the PTV, and those patients in our study receiving 40 Gy prescribed directly the GTV, it may be that these approaches yield similar dose distributions and outcomes.

**Table 5:** Summary of SBRT Reirradiation Literature

Study	N	Location		SBRT Dose (Gy)	Fx	LC	OS	Toxicity
Kelly et al. [14]	37	In-field & Out-of-field	Unspecified	40 – 50	4	92.0% (2y)	59.0% (2y)	33.0% Grade 3 0.0% Grade 4 0.0% Grade 5
Kilburn et al. [15]	33^a^	In-field	Central & Peripheral	20 – 54	1 – 10	67.0% (2y)	45.0% (2y)	3.0% Grade 3 0.0% Grade 4 3.0% Grade 5
Trakul et al. [16]	15	In-field	Central & Peripheral	20 – 50	1 – 5	65.5% (1y)	80.0% (1y)	11.6% Grade 2/3 0.0% Grade 4 0.0% Grade 5
Owen et al. [17]	18	In-field & Out-of-field	Central & Peripheral	40 – 60	3 – 10	90.0% (2y)	88.0% (1y)	0.0% Grade 3 0.0% Grade 4 0.0% Grade 5
Parks et al. [18]	27	In-field & Out-of-field	Central & Peripheral	30 – 54	3 – 5	72.0% (2y)	79.0% (2y)	25.9% Grade 3 3.7% Grade 4 0.0% Grade 5
Patel et al. [19]	26	In-field & Out-of-field	Central & Peripheral	15 – 50	3 – 5	78.6% (1y)	52.3% (1y)	0.0% Grade 3 0.0% Grade 4 0.0% Grade 5
Reyngold et al. [20]	39	In-field & Out-of-field	Unspecified	20 – 60	1 – 5	77.0% (1y)	22 mo. (med.)	10.3% Grade 3 2.6% Grade 4 0.0% Grade 5
Trovo et al. [21]	17	In-field	Central	30	5 – 6	86.0% (1y)	59.0% (1y)	23.0% Grade 3 0.0% Grade 4 11.8% Grade 5
Present Study	20	In-field	Ultra-central	25 – 45	5	30.0% (1y)	45.0% (1y)	5.0% Grade 3 0.0% Grade 4 5.0% Grade 5
				≥40	5	66.7% (1y)	77.8% (1y)	

^a^Includes patients previously treated with SBRT rather than CF-EBRT

Other radiotherapy approaches, including conventionally fractionated IMRT or proton therapy, have been employed in the recurrent setting following local treatment failure. A report from the MD Anderson Cancer Center in 2014 demonstrated the safety and feasibility of either IMRT or passive-scatter proton therapy (PSPT) in the re-irradiation setting [10]. In this study, the majority of patients were treated for in-field failures, and comparable to our experience with SBRT, the local control at 1 year was 49.2%. However, 7% and 10% of patients experienced grade 3 esophageal and grade 3 pulmonary toxicity respectively, although no differences in toxicity were noted between patients treated with IMRT or PSPT. A second report from MD Anderson, comprising 22 patients who received re-irradiation with intensity modulated proton therapy (IMPT), suggested even better results, with those patients who received a re-irradiation EQD2 of at least 66 Gy achieving 100% local control at 1 year [28]. Furthermore, although pulmonary toxicity was similar to the previous study, no grade 3 esophageal toxicity was observed. Unfortunately, there is marked patient, tumor, and treatment heterogeneity between these studies which prevents meaningful comparison, although the generally poor overall prognosis in conjunction with the short treatment time drives our preference for SBRT. Taken together, a marked dose-response seems evident, but the optimal re-irradiation technique must be tailored to the patient and may depend on the expertise and technology available at the treating center.

While promising, the current study has several limitations. Due to the relatively stringent inclusion criteria – ultra-central in-field NSCLC local recurrence without distant metastases treated with SBRT in five-fractions – there were relatively few patients reviewed over an entire decade. Furthermore, the retrospective nature of the review is limited by typical inherent pitfalls such as limited and inconsistent follow-up. Patients in the low-dose stratum tended to have larger recurrent tumors, and although this did not reach the level of statistical significance it may explain the poor local control and overall survival in this group. Furthermore, although the patients otherwise appeared to be well balanced, there may be unobserved confounding variables which biased our results. Nonetheless, our findings add to the growing body of literature suggesting the safety and efficacy of SBRT re-irradiation for NSCLC recurrence following high-dose CF-EBRT. Furthermore, these data suggest that a five-fraction regimen, with a dose of 40 Gy prescribed directly to the GTV may be an appropriate technique in order to best achieve local control. However, this treatment paradigm is aggressive, with cumulative EQD2 values exceeding 100 Gy, and patients must be counseled regarding potential morbidity and even death, particularly with doses above 40 Gy. Future prospective clinical trials are warranted to determine the optimal treatment paradigm for this patient population.

## Conclusions

In our retrospective series, we confirm the generally poor overall prognosis for patients with in-field NSCLC recurrences following CF-EBRT. However, we demonstrate the feasibility of re-irradiation utilizing a five-fraction SBRT approach, even in the setting of ultra-central tumors with abutment of the esophagus or central airway. Overall, this approach was well tolerated with an acceptable toxicity profile, and it appears that dose escalation above 35 Gy may offer improved outcomes. This approach, however, is quite aggressive and caution is warranted.
